# Most patients with an increased risk for sepsis-related morbidity or death do not recognize sepsis as a medical emergency: results of a survey study using case vignettes

**DOI:** 10.1186/s13054-023-04733-x

**Published:** 2023-11-17

**Authors:** Sebastian Born, Carolin Fleischmann-Struzek, Wiltrud Abels, Silke Piedmont, Edmund Neugebauer, Konrad Reinhart, Evjenia Toubekis, Odette Wegwarth, Daniel Schwarzkopf

**Affiliations:** 1 Institute of Infectious Diseases and Infection Control, Jena University Hospital, Stoystraße 3, 07743 Jena, Germany; 2 Center for Sepsis Control and Care, Jena University Hospital, Jena, Germany; 3 Department of Anesthesiology and Intensive Care Medicine, Charité – Universitätsmedizin Berlin, Berlin, Germany; 4 Sepsis Foundation, Berlin, Germany; 5 Brandenburg Medical School Theodor Fontane, Neuruppin, Germany; 6 Department of Emergency Medicine Campus Benjamin Franklin, Charité – Universitätsmedizin Berlin, Berlin, Germany; 7 Heisenberg Chair for Medical Risk Literacy and Evidence-Based Decisions, Clinic for Anesthesiology and Intensive Care Medicine, Charité – Universitätsmedizin Berlin, Berlin, Germany; 8 Center for Adaptive Rationality, Max Planck Institute for Human Development, Berlin, Germany; 9 Department of Anesthesiology and Intensive Care Medicine, Jena University Hospital, Jena, Germany

**Keywords:** Sepsis, Knowledge, Population at risk, Early diagnosis, Health literacy

## Abstract

**Background:**

Sepsis is a medical emergency with potentially life-threatening consequences. Patients play a crucial role in preventing and recognizing sepsis at an early stage. The understanding of risk groups’ sepsis knowledge and their ability to use this knowledge to recognize sepsis as an emergency is incomplete.

**Methods:**

We conducted a cross-sectional survey in Germany and included a sample of 740 persons stratified by age (< 60 years,  ≥ 60 years), specific chronic diseases (e.g. diabetes, chronic diseases, cancer), and region (Berlin/Brandenburg vs. other federal states of Germany). Standardized questionnaires were administered by a market research institute through online, telephone, or face-to-face methods. We assessed sepsis knowledge through a series of questions and the ability to recognize sepsis as an emergency through five case vignettes. To identify predictors of sepsis knowledge and the ability to recognize sepsis as a medical emergency, we conducted multiple linear regressions.

**Results:**

Of the 36 items on sepsis knowledge, participants answered less than 50 per cent correctly (mean 44.1%; standard deviation (SD) 20.1). Most patients knew that sepsis is a defensive host response to infection (75.9%), but only 30.8% knew that vaccination can prevent infections that lead to sepsis. Across the five vignettes, participants identified sepsis as an emergency in only 1.33 of all cases on average (SD = 1.27). Sepsis knowledge was higher among participants who were older, female, and more highly educated and who reported more extensive health information seeking behaviour. The ability to recognize sepsis as an emergency was higher among younger participants, participants without chronic diseases, and participants with higher health literacy, but it was not significantly associated with sepsis knowledge.

**Conclusions:**

Risk groups showed low levels of knowledge regarding the preventive importance of vaccination and a low ability to recognize sepsis as a medical emergency. Higher levels of sepsis knowledge alone were not sufficient to improve the ability to identify sepsis as a medical emergency. It is crucial to develop effective educational strategies—especially for persons with lower education levels and infrequent health information seeking behaviour—that not only transfer but also facilitate the choice of appropriate actions, such as seeking timely emergency care.

*Trial registration:* DRKS00024561. Registered 9 March 2021.

**Supplementary Information:**

The online version contains supplementary material available at 10.1186/s13054-023-04733-x.

## Introduction

Sepsis is a life-threatening organ dysfunction due to a dysregulated host response to an infection caused by bacteria, viruses, protozoa, fungi or parasites [1]. Globally, approximately 49 million patients are affected by sepsis every year, and 20% of global deaths are considered to be associated with sepsis [2]. COVID-19 often leads to sepsis; approximately one-third of hospitalized COVID-19 patients were found to have sepsis-related organ dysfunction [3].

The risk of infections as well as their progression into sepsis can be decreased by vaccination and adequate hygiene measures [4, 5]. Once sepsis has developed, early diagnosis and emergency treatment is required since delays in medical care increase the risk of mortality [6–9]. In addition, approximately 76% of all sepsis cases occur outside the hospital, where patients are not closely monitored by specialized health care personnel [10, 11]. Thus, patients themselves are important agents in the prevention of sepsis as well as the early recognition of signs of sepsis-related organ dysfunction. However, previous survey studies found knowledge on sepsis to be low in the general public [12–19]. Since the risk of sepsis and sepsis-related death is increased in certain groups of patients, such as elderly individuals, patients with chronic diseases, and patients with immune deficiency [20, 21], targeted educational interventions are needed to specifically address these patients. However, there is a paucity of survey studies assessing sepsis knowledge among risk groups [12, 22, 23].

In addition, evidence from patients with other medical emergencies, such as stroke, suggests that the impact of specific disease knowledge on adequate behaviour, namely seeking emergency care, is low [24, 25]. Therefore, in addition to knowledge levels, factors influencing individual competency to *apply* sepsis knowledge in medical situations, e.g. to respond to sepsis as an emergency, need to be investigated.

To address this, we conducted a survey study to investigate gaps in different domains of sepsis knowledge, the ability to recognize sepsis as an emergency and factors influencing both sepsis knowledge and the ability of recognition among specific risk groups for sepsis.

## Methods

### Study design and setting

This cross-sectional survey study was conducted between March 22 and July 8, 2021 (preregistration: DRKS00024561). The study is part of the SepsisWissen (SepWiss) intervention study (Strengthening the health literacy of patients at high risk for sepsis to improve early diagnosis and treatment of sepsis; preregistration: DRKS00024475), which aims to increase sepsis knowledge and the ability to recognize sepsis as an emergency by a multifaceted information campaign in the German states of Berlin and Brandenburg. The effectiveness of the SepWiss campaign is evaluated through a controlled pre–post-design using the other German states as the control condition. Part of the evaluation involves a longitudinal survey among risk groups. The current study reports findings from the first wave of the risk group survey (preintervention). Ethical approval for the study was obtained from the Institutional Review Board (IRB) of the Friedrich-Schiller University Jena (2020-1921-Bef). The reporting of the study followed the checklist for reporting of survey studies (CROSS) [26].

### Sample recruitment and survey conduction

Based on a sample size calculation for the evaluation of the SepWiss campaign, it was our aim to include 740 persons for the survey—370 from the intervention region (Berlin-Brandenburg) and 370 from the control region (other German federal states). Inclusion criteria were age above 18 and belonging to at least one of the sepsis-specific risk groups defined by age ≥ 60 y and pre-existing comorbidities. Since higher age is a risk factor of its own, participants with age ≥ 60 both with relevant comorbidities and without them were included. Among persons aged under 60 years, only those reporting predefined chronic diseases were included.

Since no registry or representative public data base on the respective risk groups were available, we commissioned the market research institute IPSOS to recruit participants via its network and methods, which were established in its previous healthcare market research projects. The market research institute was required to achieve predefined numbers of patients within strata defined by age (< 60 years, ≥ 60 years), specific chronic diseases (e.g. diabetes, chronic diseases, cancer), and region (Berlin/Brandenburg vs. other federal states of Germany). Explicit sample requirements are shown in Additional file 1: Table S1. The recruitment of persons without chronic diseases was realized via an existing consumer survey panel. Panel members were invited to take part in the survey, and participation was voluntary and not incentivized. For the group of persons with specific diseases, sample recruitment was complex and depending on the individual target group. It was carried out via advertising the survey to self-help groups, to general practitioners, and in social media, as well as by contacting individual participants of previous studies conducted by IPSOS. Persons who gave informed consent to participate in the survey study were first screened with respect to the predefined strata. If they met the criteria for one of the required strata, they answered a standardized questionnaire. Interviews were conducted via online, telephone or face-to-face methods. IPSOS continued recruitment until the target number of participants within each defined strata was achieved.

### Development of the questionnaire

The development of the questionnaire followed recommended methods and steps [27, 28]. First, we selected and defined constructs based on theory and previous research. Of relevance for this study were the constructs of *sepsis knowledge* [22] and the *ability to recognize sepsis as an emergency* and relevant influencing factors. We included sociodemographic information and *health information seeking behaviour* (frequency and sources of information) since their influence on sepsis knowledge has been previously shown [29]. In addition, we investigated *health literacy*—the competency to seek, interpret, and use health information—since it can be an important predictor of health behaviour and health status [30–33]. We also developed the new construct of *urgency ratings of medical situations not related to sepsis* to measure the tendency to appraise different medical situations not related to sepsis as emergencies. Second, we created an item pool and developed the first draft of the questionnaire to measure these constructs, mostly relying on preexisting validated instruments, which were partly adapted. The content validity, relevance and comprehensibility of the questionnaire were evaluated by cognitive interviews with experts in the fields of emergency medicine, infectious diseases, intensive care medicine and psychiatry and one patient (*n* = 6), and the questionnaire was revised based on the results. A pretest of the revised questionnaire was conducted in a pilot survey among members of patient support groups for chronic diseases (*n* = 71), and item characteristics as well as the number of missing responses were investigated. Based on the results of the pilot study, we modified minor details of the questionnaire to develop the final draft.

### Variables

Concerning sociodemographic data, age, sex, educational level, employment state and health insurance type were recorded. Educational level was defined by categorizing the educational degrees of Germany or the former German Democratic Republic of Germany, respectively (low: no degree or “Hauptschule”, which corresponds 8 to 9 years of school; intermediate: “Regelschule”, which corresponds to 10 years of school; high: university entrance qualification or a university degree).

#### Sepsis knowledge

We assessed four domains of sepsis knowledge using an adaptation of an existing instrument [22]: definition and epidemiology (12 items), general prevention (3 items), symptoms (7 items), and risk factors (7 items). In addition, seven items on sepsis-specific vaccination knowledge were created, including questions on vaccines that are recommended for risk groups (Influenza, Pneumococci, Meningococci, Haemophilus influenzae B, and COVID-19 vaccines) and distractors (vaccination against human papillomavirus and borreliosis). Correct answers were coded as one, and incorrect answers as well as “I don’t know” answers were coded as zero. Overall scores were calculated as sum scores (i.e. the number of correctly answered items) and mean scores (i.e. the percentage of correctly answered items) for each domain as well as for all items.

#### Ability to recognize sepsis as an emergency and urgency ratings of medical situations

Case vignettes have been used to assess the ability to recognize medical emergencies in the stroke action test [34]. Following this example, we constructed 15 case vignettes including typical medical emergencies, of which five were sepsis-related emergencies (e.g. sepsis caused by urinary tract infection, sepsis caused by respiratory infection), five were nonsepsis emergencies (e.g. heart attack or stroke), and five were nonemergencies (e.g. uncomplicated cystitis). Participants had to rate how they would react in a described medical situation using an ordinal three-point scale (0 = “Wait one day and then decide”, 1 = “Visit the general practitioner on the same day”, and 2 = “Call the emergency services or go to the emergency room immediately”). The *urgency ratings of medical situations not related to sepsis* were calculated as the mean score for the 10 case vignettes not related to sepsis. To calculate the *ability to recognize sepsis as an emergency,* the answers to the five sepsis-related vignettes were dichotomized (1 = “Call the emergency services or go to the emergency room immediately”, 0 = all other categories). Afterwards, the sum (i.e. the number of correctly solved vignettes) as well as the mean (i.e. the percentage of correctly solved vignettes) scores were calculated. For means of comparison, the same scores for an emergency rating were calculated for the other two medical situation groups not related to sepsis (nonsepsis emergencies and nonemergencies).

#### Health information seeking behaviour

For health information-seeking behaviour, we assessed (a) the *frequency of health information seeking* and (b) the *variety of health information sources*. For the frequency of health information seeking, the participants had to rate how often they searched for health information using an ordinal five-point scale ranging from “daily” to “less than once per month”. For the variety of health information sources, the participants had to indicate which of eleven given sources (e.g. consultations with a doctor or a pharmacist, newspaper, social media, self-help groups) they used to obtain health information. The number of selected health information sources was used as predictor in subsequent analyses.

#### Health literacy

From the broad concept of health literacy, we chose the domain “Disease prevention” [35] since it had the highest relevancy for our research questions. It was measured by four items from the Health Literacy Survey in Europe (HLS-EU-Q47, German version [32]). Participants had to rate how easy or difficult it was for them to access, understand, appraise, and apply information related to prevention on a four-point Likert-scale (ranging from 1 to 4). The overall *health literacy* score was calculated as the mean score of the four items.

### Analysis

Descriptive statistics were calculated for sociodemographic information as well as relevant items and scores (means and standard deviations (SDs) for metric data, frequencies and percentages for nominal and ordinal data).

**Fig. 1:**
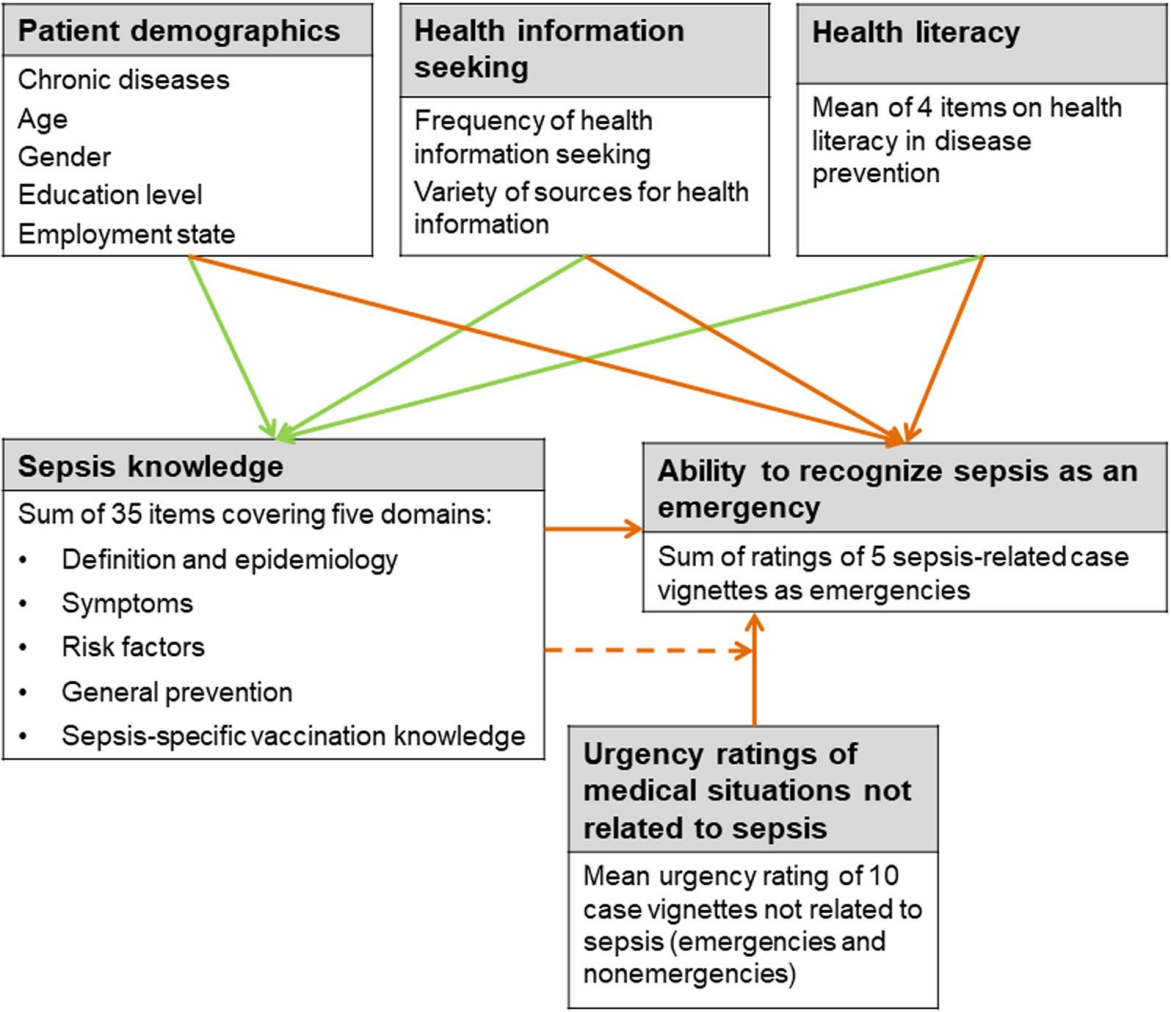
*Conceptual framework*—The study investigated influencing factors on sepsis knowledge and the sepsis emergency response. Two sets of predictive models were calculated, first to analyse predictors for sepsis knowledge (green arrows), second to analyse predictors of the ability to recognize sepsis as an emergency (orange arrows). The dashed arrow represents the interaction effect between sepsis knowledge and the urgency rating of medical situations (meaning that the size of the effect of sepsis knowledge on the sepsis emergency response depends on the urgency rating)

Figure 1 represents the conceptual framework for analyses of influencing factors. To examine the determinants of sepsis knowledge, we performed a multiple linear regression analysis. To better understand interrelated effects, we calculated several models using different sets of predictors (blockwise entry). Baseline model M1 included sociodemographic data (age, sex, education level, employment state, and health insurance type) and an indicator for the presence of at least one relevant self-reported comorbidity. Model M2 included the *frequency of health information seeking* and the *variety of health information sources* in addition to the predictors included in Model M1. In the third model (M3), *health literacy* was included in addition to the variables included in M1. The complete model (M4) contained all predictors included in Models M1–M3.

Moreover, we established several multiple linear regression models to predict the *ability to recognize sepsis as an emergency*. Models L1 to L3 included the same predictors as the respective Models M1 to M3. In addition to the variables included in Model L1, the *sepsis knowledge* score was included in Model L4. In Model L5, the *urgency ratings of medical situations not related to sepsis* were included in addition to the variables included in Model L1. The complete model (L6) included all considered variables and the interaction between sepsis knowledge and the urgency rating, since we expected that sepsis knowledge might have differing effects depending on the tendency to regard medical situations as urgent in general.

All analyses were performed using R Version 4.2.2 [36]. Regression analyses were calculated using the R-package lavaan [37]. This allowed for the implementation of full information maximum likelihood estimation to handle missing values without excluding participants [38]. To evaluate the impact of the inclusion of additional variables in comparison with the baseline models (M1 and L1), we used incremental *R*^2^ (the proportion of additional explained variance) and the chi-squared test of *R*^2^ against zero, which is used to determine whether significantly more variance is explained. We applied a significance level of *α* = 0.05.

## Results

### Participants

The market research institute was successful in recruiting the target number of *N* = 740 participants. Information on demographics and comorbidities is presented in Table 1.

**Table 1:** Demographic information and predictor variables (n = 740)

Variables	Descriptive statistics
Female gender, *n* (%)	355 (48.0)
Age in years, mean ± SD	56.6 ± 13.2
Education level, *n* (%)	
Low	174 (23.5)
Intermediate	291 (39.3)
High	275 (37.2)
Employment status, *n* (%)	
Unemployed	379 (51.2)
Employed	360 (48.7)
Health insurance, *n* (%)	
Private	86 (11.6)
Statutory	651 (88.0)
*Comorbidities*	
None, *n* (%)	197 (26.6)
At least one, *n* (%), among them	543 (73.4)
Cancer, *n* (%)	157 (28.9)
Type 1 diabetes, *n* (%)	55 (10.1)
Type 2 diabetes, *n* (%)	74 (13.6)
Chronic heart failure, *n* (%)	67 (12.3)
Chronic bronchitis, *n* (%)	70 (12.9)
Chronic renal failure, *n* (%)	1 (0.2)
Chronic liver disease, *n* (%)	47 (8.7)
Chronic neurological disease, *n* (%)	1 (0.2)
Overweight, *n* (%)	19 (3.5)
Severe rheumatic disease, *n* (%)	52 (9.6)
Severe psoriasis, *n* (%)	50 (9.2)
HIV infection, *n* (%)	81 (14.9)
Asplenia, *n* (%)	0 (0.0)
Autoimmune disease, *n* (%)	2 (0.4)
*Health Information Seeking Behaviour*	
Frequency of health information seeking, n (%)^a^	
< 1 × per month	334 (45.1)
1 × per month	158 (21.4)
1 × per week	122 (16.5)
> 1 × per week	123 (16.6)
No. of sources of health information, mean ± SD	3.5 ± 2.1
Health literacy, mean ± SD	2.68 ± 0.80
Urgency rating of medical situations, mean ± SD^b^	0.90 ± 0.33

Missing proportion of all reported variables is less than 5%

^a^For *frequency of health information seeking* the categories “daily” and “1–3 times per week” were collapsed to the category “ > 1 × per week”

^*b*^*Urgency rating of medical situations* was measured by case vignettes describing medical situations that were not related to sepsis with an urgency rating of 0—“wait another day”, 1—“visit the family physician on the same day”, and 2—“Call the emergency services or go to the emergency room immediately”

### Sepsis knowledge

Statistics for items and scores of sepsis knowledge and the ability to recognize sepsis as an emergency are given in Table 2. On average, the participants answered 44.1% (SD = 20.1) of the 36 sepsis knowledge items correctly. Most respondents correctly answered the items on the definition of sepsis.

**Table 2:** Descriptive Statistic for sepsis knowledge and the ability to recognize sepsis as an emergency

	Solved correctly by n (%) participants	Sumscore Mean ± SD	Meanscore (% correctly solved items) Mean ± SD
Sepsis knowledge (36 items)		15.88 ± 7.25	44.1 ± 20.1
Definition & Epidemiology (12 items)		5.60 ± 2.70	46.7 ± 22.5
Please decide whether the following statements regarding sepsis are true!			
Sepsis is a serious allergic reaction. (F)	336 (45.4)		
Sepsis is primarily caused by killer germs in the hospital. (F)	427 (57.7)		
Sepsis is a serious defensive reaction of the body to an infection. (T)	562 (75.9)		
Sepsis can be caused by pneumonia. (T)	187 (25.3)		
Sepsis can be caused by influenza. (T)	132 (17.8)		
Sepsis can be caused by COVID-19. (T)	150 (20.3)		
Sepsis can be caused by the spread of pathogens in the bloodstream. (T)	650 (87.8)		
Breast cancer is more common than sepsis. (F)	144 (19.5)		
More people die from heart attacks than from sepsis. (T)	75 (10.1)		
Sepsis leads to failure of vital organs, which is why sepsis patients are often treated in the intensive care unit. (T)	555 (75.0)		
Long-lasting sequelae such as chronic fatigue, kidney failure or pain are common after sepsis. (T)	311 (42.0)		
Without immediate medical treatment, the risk of dying from sepsis increases. (T)	617 (83.4)		
General prevention (3 items)		1.93 ± 0.90	64.3 ± 29.8
Please decide whether the following statements regarding sepsis are true!			
Some of the most common infections that cause sepsis can be prevented by vaccination. (T)	228 (30.8)		
Some types of sepsis can be prevented by wound and hand hygiene. (T)	564 (76.2)		
Timely response to an infection can prevent it from developing into sepsis. (T)	634 (85.7)		
Symptoms (7 items)		3.51 ± 2.08	50.1 ± 29.7
Which of the following features increase the risk of developing sepsis?			
High heart rate (T)	441 (59.6)		
Weakness in one arm or leg (F)	185 (25.0)		
Confusion or disorientation (T)	358 (48.4)		
Fever, shivering, or severe chills (T)	573 (77.4)		
Shortness of breath (T)	268 (36.2)		
Chest pain radiating to the left arm or shoulder (F)	301 (40.7)		
Extreme pain or extreme discomfort (T)	471 (63.6)		
*Risk factors (7 items)*		3.10 ± 1.71	44.3 ± 24.5
Which of the following are common warning signs of sepsis?			
Age 65 years or older (T)	346 (46.8)		
Arteriosclerosis (hardening of the blood vessels) (F)	178 (24.1)		
Smoking (T)	205 (27.7)		
Chronic diseases such as diabetes, lung diseases, cancer, kidney diseases (T)	457 (61.8)		
Weakened immune system (T)	590 (79.7)		
Veganism (pure vegetable diet) (F)	380 (51.4)		
Age younger than one year in children (T)	136 (18.4)		
Sepsis-specific Vaccination (7 items)		1.74 ± 2.05	24.9 ± 29.3
Do the following vaccinations protect against sepsis or reduce the risk of severe sepsis?			
Vaccination against human papillomaviruses (viruses that can cause benign and malignant tumours). (F)	252 (34.1)		
Vaccination against influenza viruses (T)	146 (19.7)		
Vaccination against Haemophilus influenzae B (bacteria that can cause meningitis or pneumonia) (T)	215 (29.1)		
Vaccination against pneumococcus (bacteria that can cause pneumonia, for example) (T)	207 (28.0)		
Vaccination against meningococci (bacteria which can cause, e.g. meningitis) (T)	180 (24.3)		
Vaccination against borreliosis (bacteria transmitted by ticks) (F)	132 (17.8)		
Vaccination against the Corona virus (Covid-19) (T)	158 (21.4)		
Ability to recognize sepsis as an emergency (overall)^b^		1.33 ± 1.27	26.6 ± 25.5
What would you do in the following situations or what should your loved ones do for you?			
I have the flu, feel increasingly worse and have very difficulty breathing (shortness of breath)	107 (14.5)		
The pain in the kidney area is so severe that I can hardly move. In addition, I have chills and a burning sensation when urinating	207 (28.0)		
Yesterday, I cut myself with a knife. Today, the wound looks red and inflamed. I feel very sick, weak, and can hardly leave the bed	168 (22.7)		
I have had a cough and fever for two days. Since today, I am confused and can no longer orient myself in my own living environment	368 (49.7)		
Sometime after a cat bite in the garden, I feel intense pain, am feverish, and have chills	132 (17.8)		

Missing proportion of all reported variables is less than 5%. Correct answers for the knowledge items in brackets with F = False and T = True

^a^ Sepsis knowledge was measured by items using the answer categories “yes”, “no”, “unknown”; items were dichotomized to represent the correct answer with 1, incorrect answer or unknown with 0; For domains the sumscore and the meanscore were calculated as the sum and mean of dichotomized items

^b^ Ability to recognize sepsis as an emergency was calculated based on dichotomized sepsis-related urgency ratings with 1 representing “Call the emergency services or go to the emergency room immediately” and 0 representing the other categories; the domain was calculated as the sum of the respective dichotomized items

Furthermore, the majority of patients knew that sepsis is preventable through wound and hand hygiene (76.2%), as well as a timely response to infection (85.7%). However, only 30.8% of respondents knew that some of the most common causes of sepsis can be prevented by vaccination, and only 25.3%, 17.8% and 20.3% of respondents knew that pneumonia, influenza and COVID-19 could cause sepsis, respectively.

Considering common early warning signs of sepsis, fever/shivering and severe chills were correctly identified by 77.4% of the participants. Fewer participants were aware of warning signs such as confusion/disorientation and shortness of breath (48.4% and 36.2%, respectively).

### Ability to recognize sepsis as an emergency

**Fig. 2:**
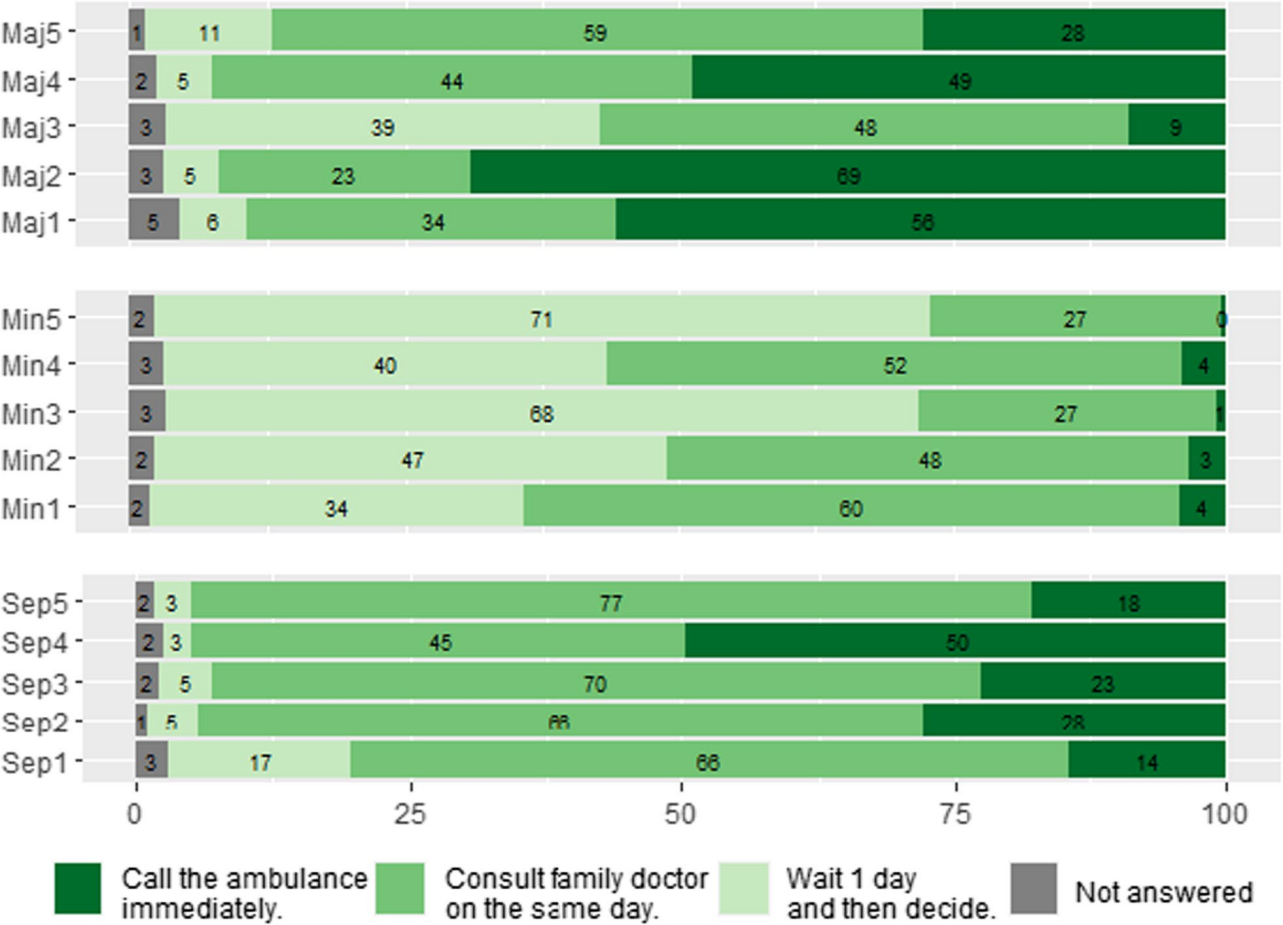
Percentages for Urgency Rating for Different Medical Situations. *Note*: Maj1—I answer the phone and sound drunk. I cannot speak clearly no matter how hard I try, but I have not drunk any alcohol. Maj2—When I get out of bed in the morning, I notice that I can no longer move my right leg and arm. Maj3 – Suddenly I cannot see anything anymore. Everything is black. After five minutes, everything is fine again. Maj4—I feel like an elephant is sitting on my chest and the feeling of pressure is getting worse. Maj5—I am told that I look pale. I feel very dizzy and it feels like my heart is fluttering and skipping beats. Min1—I have a constant urge to urinate, burning when urinating and cloudy urine. Min2—After a cat bite in the garden, I have minor swelling with surrounding redness at the bite wound, but no fever. Min3—I notice a small, round, slightly painful swelling in the area of the lower eyelid. Min4—I have pale skin and feel more exhausted than usual. I am exhausted and can do nothing more than sleep and watch TV, even though I have not done anything particularly strenuous. Min5—I have a rash on my left leg, but no other symptoms. I wonder what caused this rash. Sep1—I have the flu, feel increasingly worse and have very difficulty breathing (shortness of breath). Sep2—The pain in the kidney area is so severe that I can hardly move. In addition, I have chills and a burning sensation when urinating. Sep3—Yesterday, I cut myself with a knife. Today, the wound looks red and inflamed. I feel very sick, weak, and can hardly leave the bed. Sep4—I have had a cough and fever for two days. Since today, I am confused and can no longer orient myself in my own living environment. Sep5—Sometime after a cat bite in the garden, I feel intense pain, am feverish, and have chills

There were large differences in the urgency ratings for different case vignettes (Fig. 2). On average, participants judged only 1.33 (SD = 1.27) of the five (26.6%) sepsis-related vignettes to be medical emergencies (representing the ability to recognize sepsis as an emergency, Table 2). In comparison, 2.10 (SD = 1.43) of the five vignettes on other medical emergencies were judged as emergencies; 0.13 (SD = 0.42) of the five case vignettes on nonemergency situations were judged as emergencies.

### Predictors of sepsis knowledge

Table 3 gives an overview of the different models to predict sepsis knowledge. Sociodemographic information explained 6.3% (R^2^) of the variance in sepsis knowledge (M1). When variables on health information seeking behaviour (frequency and variety of sources) were included, additional 7.6% of the variance was explained (i.e. incremental R^2^ for M2, *p* < 0.001), including health literacy (M3) led to an additional 0.8% of the variance being explained. All predictors together explained only 14.1% of the variance in sepsis knowledge (M4). In the full model, significant predictors of sepsis knowledge were the presence of a chronic disease (*p* = 0.022), age (*p* = 0.047), female sex (*p* = 0.006), high education level (*p* < 0.001), seeking health information more often (*p* < 0.001), and using a larger variety of sources for health information (*p* < 0.001). Health literacy was a significant predictor in Model M3 but was not significant if variables on health information seeking behaviour were also included (*p* = 0.303).

**Table 3:** Prediction of sepsis knowledge

Predictors	Model 1		Model 2		Model 3		Model 4	
	*β* [95% CI]	Demographics-value	*β* [95% CI]	Demographics-value	*β* [95% CI]	Demographics-value	*β* [95% CI]	Demographics-value
*Demographics*								
Chronic disease: yes	2.428* [1.147, 3.709]	0.000	1.345* [0.111, 2.578]	0.033	2.659* [1.365, 3.953]	0.000	1.479* [0.216, 2.742]	0.022
Age (per 10 years)	0.993* [0.477, 1.509]	0.000	0.496 [− 0.016, 1.008]	0.057	1.018* [0.500, 1.535]	0.000	0.520* [0.010, 1.030]	0.047
Gender: female	2.219* [1.185, 3.253]	0.000	1.403* [0.402, 2.405]	0.006	2.170* [1.138, 3.201]	0.000	1.407* [0.406, 2.409]	0.006
Education level Ref.: low								
Intermediate	0.586 [− 0.811, 1.982]	0.411	0.542 [− 0.782, 1.865]	0.423	0.608 [− 0.773, 1.990]	0.388	0.551 [− 0.769, 1.871]	0.413
High	2.391* [0.984, 3.798]	0.001	2.388* [1.061, 3.715]	0.000	2.388* [0.995, 3.781]	0.001	2.385* [1.062, 3.708]	0.000
Employment state: employed	0.844 [− 0.340, 2.027]	0.162	0.823 [− 0.330, 1.976]	0.162	0.755 [− 0.438, 1.948]	0.215	0.788 [− 0.370, 1.946]	0.182
*Health information seeking behaviour*								
Frequency of health information seeking behaviour^a^ Ref.: < 1 × per month								
1 × per month			0.273 [− 1.033, 1.579]	0.682			0.294 [− 1.008, 1.597]	0.658
1 × per week			1.505 [0.002, 3.008]	0.050			1.532* [0.026, 3.038]	0.046
> 1 × per week			3.237* [1.674, 4.800]	0.000			3.268* [1.721, 4.814]	0.000
Variety of sources of health information			0.587* [0.305, 0.868]	0.000			0.553* [0.268, 0.838]	0.000
*Health literacy*					0.779* [0.105, 1.452]	0.023	0.341 [− 0.309, 0.991]	0.303
*R*^2^	0.063*	0.000	0.139*	0.000	0.071*	0.000	0.141*	0.000
Incremental R^2^	–		0.076*	0.000	0.008*	0.023	0.078*	0.000

Presented are the results of linear regression models to predict *sepsis knowledge*. *β*: Unstandardized Regression Coefficients. CI: Confidence Intervals. Analyses were based on *n* = 740 cases and applied full information maximum likelihood information to adjust for missing values. Four regression models were estimated to disentangle the interrelatedness of different sets of predictors. Incremental *R*^2^ measures the proportion of variance in sepsis knowledge, which is explained by the predictors in models 2 to 4 in addition to the variance explained by demographics (model 1)

^a^For *frequency of health information seeking* the categories “daily” and “1–3 times per week” were collapsed to the category “ > 1 × per week”

### Predictors of the ability to recognize sepsis as an emergency

**Fig. 3:**
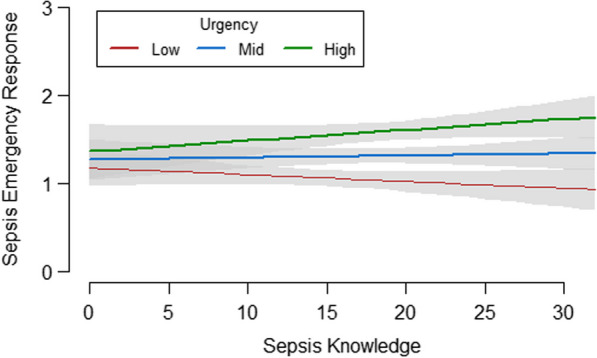
Interaction of Sepsis Knowledge and Urgency with regard to Sepsis Emergency Response

Table 4 presents the results of the models for predicting the ability to recognize sepsis as an emergency based on the case vignettes. Sociodemographic variables explained 6.3% of the variance in the emergency response (L1, *p* < 0.001). The addition of variables on health information seeking behaviour led to an increase of 3.9% in the explained variance (L2, *p* < 0.001); the addition of health literacy led to an increase of 5.9% (L3, *p* < 0.001). The addition of sepsis knowledge led to an increase of only 1.3% in the explained variance (L4, *p* = 0.025), while the addition of the urgency ratings of medical situations not related to sepsis increased the explained variance by 14.2% (L5, *p* < 0.001). The full model (L6), including all predictors, explained 23.3% of the variance in the outcome. In this model, having a chronic disease, being older, and having a higher education level predicted a lower ability to recognize sepsis as an emergency (all *p* < 0.05), while a greater variety of health information sources, higher health literacy and higher urgency ratings for medical situations not related to sepsis were associated with a higher ability to recognize sepsis as an emergency (all *p* < 0.001). Sepsis knowledge was not a significant predictor in the full model (L6, *p* = 0.717). Furthermore, there was a significant interaction between sepsis knowledge and urgency ratings of medical situations not related to sepsis (*p* = 0.007). This means that the effect of sepsis knowledge on the ability to recognize sepsis as an emergency differs depending on how urgent participants rated other medical situations not related to sepsis (Fig. 3). For participants with lower urgency ratings, sepsis knowledge had no effect on the ability to recognize sepsis as an emergency, whereas for participants with higher urgency ratings, sepsis knowledge had a positive effect on the ability to recognize sepsis as an emergency.

**Table 4:** Prediction of the ability to recognize sepsis as an emergency

Predictors	Model 1		Model 2		Model 3		Model 4		Model 5		Model 6	
	*β* [95% CI]	*p* value	*β* [95% CI]	*p* value	*β* [95% CI]	*p* value	*β* [95% CI]	*p* value	*β* [95% CI]	*p* value	*β* [95% CI]	*p* value
*Demographics*												
Chronic disease: yes	− 0.720 [− 0.983, − 0.457]	0.000	− 0.863* [− 1.124, − 0.602]	0.000	− 0.604* [− 0.864, − 0.344]	0.000	− 0.754* [− 1.017, − 0.490]	0.000	− 0.544* [− 0.781, − 0.307]	0.000	− 0.544* [− 0.790, − 0.297]	0.000
Age (per 10 years)	− 0.029 [− 0.118, 0.059]	0.516	− 0.081 [− 0.173, 0.011]	0.083	− 0.017 [− 0.101, 0.067]	0.691	− 0.043 [− 0.133, 0.047]	0.348	− 0.081 [− 0.168, 0.005]	0.065	− 0.100* [− 0.184, − 0.016]	0.020
Gender: female	0.169 [− 0.010, 0.349]	0.065	0.072 [− 0.112, 0.257]	0.442	0.145 [− 0.030, 0.319]	0.104	0.139 [− 0.041, 0.319]	0.131	0.097 [− 0.075, 0.269]	0.270	0.018 [− 0.153, 0.189]	0.834
Education level Ref.: low												
Intermediate	− 0.023 [− 0.263, 0.217]	0.851	− 0.033 [− 0.268, 0.203]	0.785	− 0.012 [− 0.248, 0.225]	0.923	− 0.031 [− 0.269, 0.207]	0.798	− 0.074 [− 0.294, 0.146]	0.508	− 0.070 [− 0.284, 0.145]	0.525
High	− 0.210 [− 0.464, 0.044]	0.105	− 0.212 [− 0.463, 0.040]	0.099	− 0.212 [− 0.459, 0.036]	0.094	− 0.243 [− 0.499, 0.013]	0.063	− 0.287* [− 0.526, − 0.048]	0.019	− 0.286* [− 0.521, − 0.051]	0.017
Employment state: employed	0.006 [− 0.200, 0.212]	0.894	− 0.003 [− 0.203, 0.197]	0.978	− 0.039 [− 0.238, 0.159]	0.699	− 0.006 [− 0.211, 0.199]	0.958	− 0.031 [− 0.226, 0.165]	0.759	− 0.069 [− 0.252, 0.114]	0.461
*Health information seeking behaviour*												
Frequency of health information seeking^a^ Ref.: < 1 × per month												
1 × per month			0.023 [− 0.224, 0.270]	0.856							0.016 [− 0.213, 0.245]	0.891
1 × per week			− 0.142 [− 0.423, 0.138]	0.320							− 0.167 [− 0.430, 0.095]	0.211
> 1 × per week			− 0.105 [− 0.439, 0.229]	0.538							− 0.202 [− 0.486, 0.120]	0.191
Variety of sources of health information			0.138* [0.083, 0.194]	0.000							0.095* [0.040, 0.150]	0.001
Health literacy					0.391* [0.285, 0.496]	0.000					0.362* [0.256, 0.468]	0.000
Sepsis knowledge							0.014* [0.002, 0.026]	0.025			0.002 [− 0.010, 0.015]	0.717
Urgency rating									1.160* [0.868, 1.452]	0.000	1.264* [0.968, 1.560]	0.000
Sepsis knowledge x urgency rating											0.050* [0.013, 0.087]	0.007
R^2^	0.063	0.000	0.102*	0.000	0.122*	0.000	0.076*	0.000	0.142*	0.000	0.233*	0.000
Incremental R^2^	–		0.039*	0.000	0.059*	0.000	0.013*	0.025	0.079*	0.000	0.170*	0.000

Presented are the results of linear regression models to predict *the ability to recognize sepsis as an emergency*. *β*: Unstandardized Regression Coefficients. CI: Confidence Intervals. Analyses were based on n = 740 cases and applied full information maximum likelihood information to adjust for missing values. Six regression models were estimated to disentangle the interrelatedness of different sets of predictors. Incremental R^2^ measures the proportion of variance in the ability to recognize sepsis as an emergency, which is explained by the predictors in models 2 to 6 in addition to the variance explained by demographics (model 1)

^a^ For *frequency of health information seeking* the categories “daily” and “1–3 times per week” were collapsed to the category “ > 1 × per week”

## Discussion

In this survey of sepsis risk groups, we found that knowledge on sepsis—especially knowledge on prevention as well as early warning signs and symptoms—was low. In addition, most patients were not able to identify sepsis as a medical emergency in case vignettes. Both health literacy and a general tendency to perceive medical situations as urgent significantly predicted the ability to recognize sepsis. Sepsis knowledge had only a small effect, which was nonsignificant after adjusting for other predictors. Interestingly, sepsis knowledge showed a larger effect on the ability to recognize sepsis as an emergency among participants with a higher tendency to rate medical situations not related to sepsis as urgent.

While 76% of participants in our study knew that sepsis is caused by the response of the body to an infection that damages the host organs and tissues, only a minority knew that sepsis is always caused by an infection and that vaccinations may help to prevent sepsis. Such knowledge gaps were also found in previous studies, such as a representative survey of the German elderly population as well as a recent representative survey in Canada [22, 39]. Similar to our results, a recent systematic review found that the knowledge on common symptoms and risk factors of sepsis was low in the public and among patients [12]. This, in combination with the poor understanding that sepsis needs to be treated as an emergency, may lead to patients delaying medical care [16]. The problem is further exaggerated by the fact that also many healthcare professionals are not familiar with sepsis and its warnings signs, causing further delays of appropriate treatment and thereby preventable deaths [12, 40].

Only a few previous studies have investigated predictors of sepsis awareness or knowledge—mostly only including patients’ demographic characteristics and socioeconomic status. Similar to most of these studies, we found that female sex [14, 17, 19, 22] and higher education levels [14, 17–19] were associated with higher sepsis knowledge. In addition, we found that participants with higher risk—namely, older persons and persons with chronic conditions—and persons with more extensive health information-seeking behaviour had higher sepsis knowledge. There were some interesting discrepancies when investigating predictors of the ability to recognize sepsis as an emergency. Here, participants who were at increased risk (older age, with chronic disease) showed a lower tendency to appraise sepsis vignettes as emergencies. We hypothesize that patients with more comorbidities may have had a higher habitation to medical symptoms and therefore did not regard the described situation as threatening. The strongest predictor of the ability to recognize sepsis as an emergency was the urgency ratings of other medical situations not related to sepsis. This measure might result from numerous factors influencing the decisions of patients to seek immediate help for a medical condition. These factors involve not only different thresholds of when symptoms are perceived as serious or worrisome but also social cognitive factors such as prioritizing everyday duties, the sense of control, the availability of support from family and friends, and expectations about health care services [41–44].

Surprisingly, higher sepsis knowledge was not essential for correctly appraising the case vignettes. Therefore, possessing illness-specific information is not equivalent to correctly applying it in real-world situations. Likewise, health literacy, which is regarded as a set of skills not only to access but also to understand, appraise and apply information for the purpose of achieving or maintaining health [45], was a significant predictor of correctly identifying sepsis vignettes as emergencies. In addition, we found an interaction effect between the urgency ratings of other medical situations not related to sepsis and sepsis knowledge. This indicates that the higher the tendency to seek timely medical help in general is, the higher the effect of specific sepsis knowledge. Therefore, it might also be important to overcome reluctance to seek timely medical help in general.

Our study provides important lessons for sepsis awareness campaigns, which should foster the understanding of infection, sepsis, and their preventability by vaccination, the early warning signs for the progression of an uncomplicated infection to sepsis and that sepsis must be managed as an emergency. Stressing the fact that vaccinations also prevent sepsis was also shown to positively impact vaccine hesitancy according to previous research [46]. Public health interventions also need to develop better strategies to reach persons with lower education levels or infrequent health information seeking behaviour. This includes, for example, campaign messaging in plain language, which is accessible to the intended audience [47], as well as pretesting information material and adapting them to the informational needs of different target groups [48]. On the other hand, given that theoretical knowledge on sepsis alone may not drive the seeking of emergency medical treatment, public health interventions must identify and use new methods to increase procedural knowledge and the ability to judge experienced symptoms. In stroke warning campaigns, the most effective campaign design was a theory-based strategy with a focus on role modelling, showing stroke survivors in the community and advertising their successful recovery after thrombolysis [49]. Following this approach, stroke survivors demonstrated that an immediate response to stroke symptoms resulted in a better outcome [50]. Such strategies may also be of potential benefit for sepsis campaigns, but further research is needed to identify effective campaign designs and messages specific to sepsis as a condition with unspecific early symptoms that is complex to recognize. In addition, barriers to seeking medical help in medical emergencies such as sepsis need to be removed. The current discussion in the German health care system mostly focuses on how to prevent patients without serious health issues from using emergency care. Unfortunately, it often neglects the unknown number of patients with serious health conditions who suffer from delays in seeking and receiving appropriate medical support. If doubt exists, it should not be left to patients or any relatives to decide whether they have a medical condition requiring emergency treatment; either the family physician, nonemergency medical on-call service, or emergency medical service should be contacted. Our results support the call for an integrated medical on-call service involving telemedicine, validated diagnostic algorithms and well-trained providers to provide 24/7 support for patients to receive appropriate and timely care as indicated by their medical condition [51].

### Strengths and limitations

This study has several strengths. It is the first investigation of sepsis knowledge in a large sample covering the broad spectrum of risk groups [12]. This study assessed the relevant different domains of sepsis knowledge and used a more complex questionnaire and analytic strategy to investigate influencing factors compared to previous studies. It is also the first study to use case vignettes to assess the ability to recognize sepsis as an emergency, which is closer to real-world behaviour than solely measuring passive sepsis knowledge. The case vignettes can be used in further studies to evaluate the effect of public health interventions.

Our study also has limitations. Since it used a convenience sample, the generalization of the results to the German population is impaired. In addition, since higher levels of public awareness of sepsis were reported for Germany in comparison with other countries [13], the international generalizability also might be limited. The case vignettes were newly developed. While we assured the content and face validity of the vignettes through extensive pretesting among both clinical experts and patients, empirical evidence for the validity to represent how patients actually would react in real-world situations cannot be provided. On the other hand, we believe there is no feasible study method to provide such empirical evidence for case vignettes on emergency situations. We believe our results provide important clues regarding the complex decision-making process in emergency situations, but we were not able to completely explain differences. Future survey studies should investigate additional possibly influencing factors like expectations to medical services, social support, as well as psychological factors like sense of control [41–44]. In addition, qualitative interviews with sepsis survivors, their relatives, and emergency health care personnel could provide further insights. Regional socioeconomic deprivation has been shown to be associated with increased sepsis incidence [52]. It could also influence sepsis knowledge and the willingness to seek acute and emergency medical care. Therefore, future studies should include aspects of regional socioeconomics and health care availability.

## Conclusions

Despite sepsis being a devastating medical emergency, the knowledge and awareness of sepsis among risk populations is still low in many aspects. In addition, most patients do not regard sepsis as an emergency when confronted with sepsis-related case vignettes, and having theoretical sepsis knowledge alone is likely not associated with making the right decisions in emergency situations. Therefore, awareness campaigns need to develop effective educational strategies, which are tailored to specific risk groups—especially persons with lower education or less intrinsic interest in health-related information. In addition, new methods need to be developed to not only transfer information but to enhance the competency to identify life-threatening emergency situations and to promptly seek medical care. Acute and emergency care systems should not leave it to patients to decide on the urgency of their medical needs, but should provide integrated on-call services with 24/7 immediate availability provided by well-trained staff.

### Supplementary Information

**Additional file 1:** Supplementary Table and Questionaire.

## Data Availability

The datasets used and/or analysed during the current study are available from the corresponding author on reasonable request. Access to the anonymized data might be granted following review and permission by the ethics commission and data protection officer of the Jena University Hospital.
