# Supplementary material for: Most patients with an increased risk for sepsis-related morbidity or death do not recognize sepsis as a medical emergency: results of a survey study using case vignettes
Source: Crit Care. 2023 Nov 17;27:446. doi: 10.1186/s13054-023-04733-x (PMC10655489; doi:10.1186/s13054-023-04733-x)
Supplement: Supplementary file 1 — Additional file 1: Supplementary Table and Questionaire. [file 13054_2023_4733_MOESM1_ESM.docx]

**Supplement**

**Supplementary Table S1**

*Pre-set criteria for the recruitment of participants with and without pre-existing diseases and actual frequencies in the sample (in brackets).*

|  | **Region Intervention** | | **Region Control** | |  |
| --- | --- | --- | --- | --- | --- |
|  | **Age < 60** | **Age ≥ 60** | **Age < 60** | **Age ≥ 60** | |
| **Cancer** |  |  |  |  |  |
| Blood cancer | 19 (19) | 7 (7) | 19 (19) | 7 (7) |  |
| Urological cancer | 4 (6) | 2 (3) | 4 (5) | 2 (3) |  |
| Breast cancer | 4 (8) | 2 (6) | 4 (9) | 2 (3) |  |
| Lung cancer | 4 (6) | 2 (4) | 4 (4) | 2 (4) |  |
| Colon cancer | 4 (5) | 2 (6) | 4 (6) | 2 (4) |  |
| Skin cancer | 4 (5) | 2 (6) | 4 (7) | 2 (5) |  |
|  |  |  |  |  |  |
| **Chronic Diseases** |  |  |  |  |  |
| Diabetes type I | 17 (24) | 3 (4) | 17 (23) | 3 (4) |  |
| Diabetes type II | 3 (7) | 17 (31) | 3 (7) | 17 (29) |  |
| Chronic cardiovascular diseases | 20 (22) | 7 (12) | 20 (23) | 7 (10) |  |
| Chronic lung diseases | 20 (25) | 7 (10) | 20 (25) | 7 (10) |  |
| Chronic kidney disease | 15 (22) | 5 (8) | 15 (19) | 5 (7) |  |
| Chronic liver diseases | 15 (17) | 5 (7) | 15 (16) | 5 (7) |  |
|  |  |  |  |  |  |
| **Auto-immune diseases** |  |  |  |  |  |
| Severe rheumatism | 14 (15) | 6 (11) | 14 (16) | 6 (10) |  |
| Severe psoriasis | 14 (16) | 6 (9) | 14 (17) | 6 (8) |  |
|  |  |  |  |  |  |
| **HIV infection** | 28 (29) | 12 (12) | 28 (28) | 12 (12) |  |
|  |  |  |  |  |  |
| **Persons with specific diseases** | **185** | **85** | **185** | **85** |  |
| **Persons without specific diseases** | **0** | **100** | **0** | **100** |  |
| **Total** | **370** | | **370** | |  |

*Note:* Blood cancer includes leukaemia, Hodgkin's disease, non-Hodgkin lymphomas. Urological cancer includes cancer of the bladder, kidney, or urinary tract. Because of multi-morbid patients, the sum of actual frequencies in the sample do not correspond to the number of persons with specific disease. Intervention-Region = Berlin/Brandenburg. Control-Region = other federal states of Germany

**Questionnaire**

1. How old are you?
2. What is your gender?
3. In which federal state do you live?
4. What is your highest level of education?
5. Are you currently suffering or have you suffered from cancer in the last 5 years?
6. What cancer do you suffer or have you suffered from?
7. What chronic diseases do you suffer from?
8. How often do you find out about health topics (excluding Corona)?
9. What sources do you use when you inform yourself about health topics?
10. How easy/difficult is it for you to...

- … find information about how to avoid or deal with certain health risks?
- … understand why you need screenings?
- … assess which vaccinations you may need?
- … decide how to protect yourself from illness based on advice from family and friends?

1. What would you do in the following situations or what should your loved ones do for you?
   1. I have the flu, feel increasingly worse and have very difficulty breathing (shortness of breath). [Sepsis-related emergency #1]
   2. I answer the phone and sound drunk. I cannot speak clearly no matter how hard I try, but I have not drunk any alcohol. [Nonsepsis emergency #1]
   3. I have a constant urge to urinate, burning when urinating and cloudy urine. [Nonemergency #1]
   4. When I get out of bed in the morning, I notice that I can no longer move my right leg and arm. [Nonsepsis emergency #2]
   5. After a cat bite in the garden, I have minor swelling with surrounding redness at the bite wound, but no fever. [Nonemergency #2]
   6. The pain in the kidney area is so severe that I can hardly move. In addition, I have chills and a burning sensation when urinating. [Sepsis-related emergency #2]
   7. I notice a small, round, slightly painful swelling in the area of the lower eyelid. [Nonemergency #3]
   8. Yesterday, I cut myself with a knife. Today, the wound looks red and inflamed. I feel very sick, weak, and can hardly leave the bed. [Sepsis-related emergency #3]
   9. Suddenly I cannot see anything anymore. Everything is black. After five minutes, everything is fine again. [Nonsepsis emergency #3]
   10. I have pale skin and feel more exhausted than usual. I am exhausted and can do nothing more than sleep and watch TV, even though I have not done anything particularly strenuous. [Nonemergency #4]
   11. I feel like an elephant is sitting on my chest and the feeling of pressure is getting worse. [Nonsepsis emergency #4]
   12. I have had a cough and fever for two days. Since today, I am confused and can no longer orient myself in my own living environment. [Sepsis-related emergency #4]
   13. I am told that I look pale. I feel very dizzy and it feels like my heart is fluttering and skipping beats. [Nonsepsis emergency 5]
   14. I have a rash on my left leg, but no other symptoms. I wonder what caused this rash. [Nonemergency #5]
   15. Sometime after a cat bite in the garden, I feel intense pain, am feverish, and have chills. [Sepsis-related emergency #4]
2. Sepsis is also known as blood poisoning. In the next section, you will be asked to assess the extent to which certain statements about sepsis (blood poisoning) are true. We will always use the term "sepsis" in the following questions.

Please decide whether the following statements regarding sepsis are true!

- 1. Sepsis is a serious allergic reaction.
  2. Sepsis is primarily caused by killer germs in the hospital.
  3. Sepsis is a serious defensive reaction of the body to an infection.
  4. Sepsis can be caused by pneumonia.
  5. Sepsis can be caused by influenza.
  6. Sepsis can be caused by Covid-19.
  7. Sepsis can be caused by the spread of infectious agents in the bloodstream.
  8. Breast cancer is more common than sepsis.
  9. More people die from a heart attack than from sepsis.
  10. Sepsis leads to the failure of vital organs, which is why sepsis patients are often treated in intensive care.
  11. Long-lasting secondary diseases such as chronic fatigue, kidney failure or pain are common after sepsis.
  12. Without immediate medical treatment, the risk of dying from sepsis increases.
  13. Some of the most common infections that cause sepsis can be prevented by vaccination.
  14. Some types of sepsis can be prevented by wound and hand hygiene.
  15. By reacting in time to an infection, it is possible to prevent sepsis from developing.

1. Which of the following features increase the risk of developing sepsis?
   1. Age: 65 years or older
   2. Arteriosclerosis (vascular calcification)
   3. Smoking
   4. Chronic diseases such as diabetes, lung disease, cancer, kidney disease
   5. Weakened immune system
   6. Veganism (purely plant-based diet)
   7. Age younger than one year in children
2. Which of the following are common warning signs of sepsis?
   1. High heart rate
   2. Weakness in an arm or leg
   3. Confusion or disorientation
   4. Fever, shivering, or severe feeling of cold
   5. Shortness of breath
   6. Chest pain radiating to the left arm or shoulder
   7. Extreme pain or discomfort
3. Do the following vaccinations protect against sepsis or reduce the risk of severe sepsis?
   1. Vaccination against human papillomaviruses (viruses that can cause benign and malignant tumors)
   2. Vaccination against influenza viruses
   3. Vaccination against Haemophilus influenzae B (bacteria that can cause meningitis or pneumonia)
   4. Vaccination against pneumococci (bacteria that can cause pneumonia, for example)
   5. Vaccination against meningococci (bacteria that can cause meningitis, for example)
   6. Vaccination against Lyme disease pathogens (tick-borne bacteria)
   7. Vaccination against the corona virus (Covid-19)
